# Patients’ perspectives on BETTER 2 prevention and screening: qualitative findings from Newfoundland & Labrador

**DOI:** 10.3399/bjgpopen17X101037

**Published:** 2017-10-04

**Authors:** Nicolette Sopcak, Carolina Aguilar, Candace I J Nykiforuk, Mary Ann O’Brien, Kris Aubrey-Bassler, Richard M Cullen, Melanie Heatherington, Eva Grunfeld, Donna P Manca

**Affiliations:** 1 Qualitative Research Lead, Department of Family Medicine, Faculty of Medicine, University of Alberta, Edmonton, AB, Canada; 2 Health Program Coordinator, Department of Family Medicine, Faculty of Medicine, University of Alberta, Edmonton, AB, Canada; 3 Associate Professor and Program Director, Health Promotion & Socio-behavioural Sciences, School of Public Health, University of Alberta, Edmonton AB, Canada; 4 Assistant Professor, Department of Family and Community Medicine, Faculty of Medicine, University of Toronto, Toronto ON, Canada; 5 Associate Professor & Director, Primary Health Care Unit, Faculty of Medicine, Memorial University of Newfoundland, St John’s, NL, Canada; 6 Research Coordinator, Primary Health Care Unit, Faculty of Medicine, Memorial University of Newfoundland, St John's, NL, Canada; 7 Study Coordinator, Department of Family Medicine, Faculty of Medicine, University of Alberta, Edmonton, AB, Canada; 8 Director, Knowledge Translation Research, Health Services Research Program, Ontario Institute for Cancer Research, Toronto ON, Canada; 9 Giblon Professor and Vice-Chair (Research), Department of Family and Community Medicine, Faculty of Medicine, University of Toronto, Toronto ON, Canada; 10 Associate Professor and Research Director, Department of Family Medicine, Faculty of Medicine, University of Alberta, Edmonton, AB, Canada

**Keywords:** patients, chronic disease, primary prevention, primary care

## Abstract

**Background:**

Chronic disease prevention and screening (CDPS) has been identified as a top priority in primary care. However, primary care providers often lack time, evidence-based tools, and consistent guidelines to effectively address CDPS. Building on Existing Tools to Improve Chronic Disease Prevention and Screening in Primary Care (BETTER) is a novel approach that introduces a new role, that of the prevention practitioner; the prevention practitioner meets with patients, one on one, to undertake a personalised CDPS visit. Understanding patients’ perspectives is important for clinicians and other stakeholders aiming to address and integrate CDPS.

**Aim:**

To describe patients’ perspectives regarding visits with a prevention practitioner in BETTER 2, an implementation study that was carried out after the BETTER trial and featured a higher proportion of patients in rural and remote locations.

**Design & setting:**

Qualitative description based on patient feedback surveys, completed by patients in three primary care clinics (urban, rural, and remote) in Newfoundland and Labrador, Canada.

**Method:**

Patients’ perspectives were assessed based on responses from 91 feedback forms. In total, 154 patients (aged 40–65 years) received ≥1 prevention visit(s) from a prevention practitioner and were asked to provide written feedback. In addition to demographics, patients were asked what they liked about their visit(s), what they would have liked to be different, and invited to make any other comments. Qualitative description was used to analyse the data.

**Results:**

Four main themes emerged from patients’ feedback: value of visit (patients appreciated the visit with a prevention practitioner); visit characteristics (the visit was personalised, comprehensive, and sufficiently long); prevention practitioners' characteristics (professionalism and interpersonal skills); and patients’ concerns (termination of the programme and access to preventative care).

**Conclusion:**

Patients appreciated the visits they received with a prevention practitioner and expressed their desire to receive sustained CDPS in primary care.

## Introduction

Primary care providers have increasingly adjusted their focus to CDPS to respond to the steady increase of chronic conditions in Canada such as diabetes, heart disease, and cancer.^1,2^ However, primary care providers often lack time, evidence-based tools, and consistent guidelines to effectively address CDPS. BETTER, a pragmatic cluster randomised controlled trial, demonstrated that the addition of individualised prevention visits with prevention practitioners significantly improved patient CDPS outcomes for cardiovascular disease, diabetes, cancer, and associated lifestyle factors (diet, physical activity, smoking, and alcohol) in participating urban settings.^3 ^The key component of BETTER, the prevention practitioner, is typically a member of a practice team (for example, a nurse practitioner, nurse, licensed practical nurse, or dietician) who meets with patients, one on one, to develop a personalised 'prevention prescription' using the BETTER tools,^4^ the BETTER approach,^5^ and brief action planning.^6^ BETTER 2, an implementation study and second iteration of BETTER following the BETTER trial, included a higher proportion of Aboriginal Canadian patients and those in rural and remote locations, compared with those who participated in BETTER.^7^

Details about the BETTER approach have been published previously.^3–5,7,8^ Implementation of the approach was studied in diverse settings including rural and remote practices.^5,9^ The quantitative results of the implementation of BETTER 2, which include descriptive statistics, are currently under review for publication. A qualitative implementation study identified five key elements that hindered or facilitated the implementation of BETTER 2 (Box 1).^9^

Based on focus groups and key informant interviews with primary care providers, researchers, administrators, and other community partners, the Consolidation Framework for Implementation Research (CFIR) was used to frame the results systematically by applying key domains that are considered most salient in programme implementation. The evaluation of the qualitative implementation revealed that some physicians perceived the 1-hour visit with a prevention practitioner to be a barrier for patients:

*'One of the barriers* [is that] *some of my patients are hard to get to do preventive care *[...] *it’s because they have to take the bus up to the screening centre* […] *They take a bus to come see me or they walk; they don’t drive, so they’re my patients that are less likely to come in for an hour and see a practitioner* […] *I mean there’s those barriers. There’s financial barriers for patients, and time.'*(Physician 3)

Other physicians indicated that the patients who would go to have the prevention visit were those who did not really need it. For instance, one doctor stated that:

'*Most of the people in my practice that don’t need to go, they are the ones that go. I think* [the prevention practitioner] *is getting the “worried well”.'* (Physician 10)

Despite the outcome measures, descriptive statistics, and the input from participants in the BETTER 2 programme, one perspective was missing: that of patients. What did patients think of their visits with a prevention practitioner? Did those who received this perceive it as a duplication of services or unnecessary? Did they like it? If they did, what did they like about it? What would they have liked to be different? The literature suggests that patients often have a different view of their needs than their primary care providers.^10^ Understanding patients’ perspectives is important for clinicians and other stakeholders aiming to address and integrate CDPS; as such, this study aimed to describe patients’ perspectives of the BETTER approach and the prevention visit(s) they received with a prevention practitioner as part of the BETTER 2 programme.

## Method

### Participants and setting

This study is a small component of a larger programme evaluation of the BETTER 2 programme (described elsewhere).^7^ Three primary care settings (urban, rural, and remote) in Newfoundland and Labrador, Canada, participated in the implementation of the BETTER 2 programme. Three prevention practitioners were trained to deliver the programme using the BETTER 2 Toolkit,^4^ which included:

- blended care paths for CDPS and associated lifestyle risk factors; and
- brief action planning,^6^ a structured approach to behaviour change based on the principles of motivational interviewing.

The role of prevention practitioner was taken on by a licensed practical nurse in the rural site, a nurse practitioner shared between two small remote communities, and a nurse practitioner in an urban academic practice.

### Patients and recruitment

Waiting-room posters, clinician referral, the media (for example, news articles), and mail-out invitations were used to invite patients aged 40–65 years to enroll in BETTER 2. Interested patients contacted their clinic to receive more information about the study and schedule a prevention visit with the prevention practitioner. Patients provided written informed consent at their first visit and were invited to attend a follow-up visit with the prevention practitioner approximately 6 months later. After each prevention visit (baseline and follow-up), patients were provided with a feedback form and information letter, which invited them to give the study team feedback on the programme and their prevention visit(s).

Providing feedback was voluntary and was completed by submitting﻿ anonymous forms using a closed comment box located in each clinic’s waiting area or through the mail using a pre-addressed, stamped envelope. Patients were made aware that submitting their completed feedback form to the study team indicated that they were consenting to participate in that component of the study.

**Box 1.** Facilitators and barriers of the BETTER 2 programme, described using the CFIR.^9^

CFIR domain	Key element	Barrier	Facilitator
**Intervention characteristic**	Complexity	Amount of material is overwhelming and time consuming	Strong evidence base (previous RCT) Patients liked comprehensiveness (multifactorial approach, as opposed to specific disease or organ)
	Cost	Intervention too costly	Intervention is cost effective (investing in prevention offsets acute care costs)
**Outer setting**	Perception of fit	Lack of remuneration Lack of resources (particularly staff) Physicians’ perception that prevention practitioner's visit duplicates services	Other stakeholders (including managers) see CDPS as a 'hot topic' Patients see visits as valuable, necessary, and& motivating
**Characteristics of individuals**	Prevention practitioners	None	Interest in prevention Ability to support and motivate patients
**Inner setting**	Local champion	Lack of local champion or losing a local champion (for example, physician left community)	None
	Working *in* a team versus working *as* a team	Not working as a team (for example, team tensions, lack of relationship, competition, or unclear roles)	Working as a team (for example, trust or physicians appreciating prevention practitioners structuring CDPS)
**Process**	Planning and engaging	Not including collaborators enough in planning process	Starting collaborative conversations early
	Collaboration and teamwork	Lack of awareness/misconception of BETTER approach	Team members being available Frequent and open conversations

CDPS = chronic disease prevention screening. CFIR = Consolidation Framework for Implementation Research. RCT = randomised controlled trial.

### Design and intervention

Patients were initially invited to participate in telephone interviews, but only two patients over the course of 2 months participated. These two patients gave positive reviews of BETTER 2 and the team recognised that there was a risk of selection bias as patients who were unhappy were less likely to participate in interviews. Given the low response to requests for telephone interviews, and as patients had already committed a significant amount of time to the project (approximately 3 hours per visit including filling out surveys, travel time, and so on), the team designed a patient survey to obtain feedback. Patients who completed at least one visit with a prevention practitioner were invited to fill out a feedback form that was designed to take around 5–7 minutes to complete. 

Questions on the feedback form aimed to ascertain:

- patients' demographic details;
- how they heard about the prevention visits;
- the number of visits they had attended;
- what they liked about their visit;
- what they would have liked to be different about their visit; and
- any other comments.

### Data analysis

Survey responses were collected and sorted by question in Microsoft^®^ Excel. Two investigators with expertise in qualitative methods performed a first iteration of data analysis independently using content analysis and qualitative description. The independent analyses were then reviewed and discussed by the same researchers, along with two other members of the larger team to ensure consensus. Minor discrepancies were resolved through discussion.

## Results

In total, 154 patients (35 male [23%] and 119 female [77%]) participated in the BETTER 2 programme. A total of 91 feedback forms were returned, of which 26 (29%) were from men and 65 (71%) from women. Thirty-nine (43%) of responses were from the remote/rural areas and 52 (57%) from the urban setting.

Four main themes emerged from the patient feedback:

- value of visit;
- visit characteristics;
- prevention practitioner characteristics; and
- patients’ concerns.

### Value of visit

All patients who provided feedback described their visit(s) with a prevention practitioner positively, indicating that they were valuable. Patients commented on the visit in general by expressing their gratitude, and articulating their appreciation of the programme:

*'It was nice to have someone look at the big picture regarding my health and develop a plan for me to go forward. Wish I had someone look from a preventative nature long before this. Bravo!'* (Patient 11)

*'This came at an important time in my life. Excellent project! Made me feel better about my health. An awesome foundation to becoming healthier.'* (Patient 32)

*'As I get older, I seem to be more and more concerned about my health. This type of programme provides me with information to know what I can do to be less concerned. Thank you.'* (Patient 2)

### Visit characteristics

The first theme described how patients valued their prevention visits or the programme overall, whereas the second emerged from patients’ specific feedback about the actual prevention visit. Patients commented on the characteristics of the visit, such as having enough time available (not feeling rushed by the visit), and it being one on one and personalised to their specific needs:

*'Uninterrupted one-to-one time with a healthcare professional who didn’t rush things. Everything was specific to my healthcare needs, not just general teaching.'* (Patient 40)

*'Visits were one on one. Lots of discussion about health, ageing, diet and exercise. Very friendly practitioner, no stress atmosphere.'* (Patient 70)

*'Thank you for the opportunity to learn about the cause and effect impacts of lifestyle, especially diet and exercise on health in a very personalised way.'* (Patient 86)

### Prevention practitioner characteristics

Patient feedback about the prevention practitioners was overwhelmingly positive. Comments regarding the characteristics of the prevention practitioners were grouped into two main themes:

- professionalism; and
- interpersonal skills.

Although some patients’ comments focused on one theme, others were associated with both.

#### Professionalism

Patients commented on prevention practitioners’ professionalism, describing them as knowledgeable, offering good advice, and being well prepared before, and during, the visit:

'[The prevention practitioner] *was very professional in her approach. She was very organised. She took* [the] opportunity *for teaching and made appointments as needed for follow-up on topics needing attention. I came away determined to meet my goals to stay healthy.'* (Patient 10)

*'She went over my chart beforehand and was prepared with regards to my medical history. She was very polite, easy to understand, and was easy to talk to. She was motivating!'* (Patient 13)

*'She was very professional. She gave me lots of information (more than the doctors).'* (Patient 58)

*'She was very thorough and knew my medical history, which is complex, prior to my visit. Felt like she knew what she was doing.'* (Patient 75)

#### Interpersonal skills

Patients also remarked on prevention practitioners’ interpersonal skills, perceiving the practitioners to be personable, motivating, and caring. Patients also expressed that they enjoyed the relaxed atmosphere that prevention practitioners created and appreciated being listened to:

*'Personal, not rushed. She listened. She provided information that was relevant to me.' (Patient 55)*

*'Friendly, non-judgmental. Assisted me with problem solving around my issues and helped me establish attainable and measurable goals within a set timeline. Enthusiastic, encouraging.'* (Patient 61)

*'The young nurse has a way of cutting through the bull and getting to the point — yet, in a caring, gentle way.'* (Patient 85)

*'The* [prevention practitioner] *was very knowledgeable and it was a pleasure to go to these visits as she was very personable — often doctors, due to their demands I guess, do not provide this atmosphere.'* (Patient #86)

#### Patients’ concerns

Although patients’ feedback was positive regarding their prevention visits and the prevention practitioners, they also voiced concerns, which emerged as:

- limited follow-up and a desire to continue with the programme; and
- limited access to preventative care.

##### Limited follow-up and the desire to continue with the programme

Patients suggested that the follow-up visit should be conducted earlier, as many perceived the duration between the first and second visits (6 months) as too long. Moreover, they expressed their desire to continue with the programme and their disappointment that it ended:

*'I would have liked for someone to follow-up in between both visits. It would have reminded me a little more and maybe helped push me a little more in regards to the goals I set.'* (Patient 27)

*'I would like to continue. This was a worthy concept —﻿ sadly no longer implemented in Labrador.'* (Patient 31)

*'I would like to continue. I put a lot of effort into making things change and they did. Now what? It is too difficult to see a doctor.'* (Patient 33)

##### Limited access to preventative care

Although not specific to the prevention visit or the BETTER approach, patients commented on their perception that there is limited access to preventative care within the current healthcare system. Specific suggestions included an expansion of the programme to include patients of all ages, and for prevention visits to be part of a yearly routine. Some patients also expressed their overall frustration with the lack of prevention and screening available in their setting:

*'It would be beneficial to start earlier, at an earlier age so it becomes a natural yearly routine, especially to improve on addictions and overweight issues.'* (Patient 87)

*'With the ageing population of our citizens, the general poor health in such a rich/wealthy country, this type of programme, if pursued and pushed more and even for older people … I think would in the long run, save healthcare costs.'* (Patient 85)

*'This programme should be more readily available on a more ongoing basis, but if it is impossible for doctors specifically to implement these methods, a more 'team effort' should be applied between doctor and practitioner as, since these visits, my doctor never spoke of additional preventative/changes that should be instated to improve my overall health. These methods should be implemented in the medical community as it would, in my opinion, promote a healthier society as a whole.'* (Patient 86)

### Triangulation with results from qualitative implementation study

The qualitative implementation study identified that, although primary care providers (including prevention practitioners) and managers appreciated and embraced the prevention visits, some physicians questioned the value of such visits for patients.^9^ Interestingly, none of the patients who provided feedback shared those doubts, despite having the opportunity do to so anonymously.

The three prevention practitioners in this project (two nurse practitioners and one licensed practical nurse) perceived the prevention visits to be valuable and saw them as a good fit with their role as health professionals and their focus on prevention. As one practitioner commented:

*'It brings attention to the fact of prevention, you know? And that’s what the project is about, and I mean that’s what nursing is. That’s what I do, right? You know, so I like it, I really, really do.'* (Physician 3)

Moreover, the prevention practitioners participating in BETTER 2 commented that they integrated some of the tools and techniques (for example, use of BETTER algorithm or brief action planning) into their regular practice outside of the BETTER 2 study.^9^

## Discussion

### Summary

This study focused on patients’ perspectives of the prevention visits they received as part of the BETTER 2 study, which they perceived as beneficial, important, and meaningful. Patients also expressed their concern about the termination of the programme and their having limited access, as they saw it, to preventative care in their settings.

### Strengths and limitations

Patients’ perspectives contribute a critical layer to the growing body of research on CDPS in primary care across urban and rural/remote contexts. Given that patients are getting more involved in the healthcare decision-making process, their experiences and perceptions are important. The authors' ability to collect 91 patient feedback forms, from a potential total of 154, is a major strength of this study; it demonstrates that a large proportion of participating patients took advantage of the opportunity to share their perspectives anonymously. Feedback was also obtained from diverse settings; 43% of completed feedback forms came from rural/remote primary care settings and 57% from urban settings. In the context of the larger study — in which patients were asked to dedicate time to the prevention visits (baseline and follow-up), completion of health surveys, and any resulting laboratory or screening tests when these were out of date — feedback forms were a non-intrusive, quick, and an easy way to collect patients’ comments and opinions.

One limitation of this qualitative study is that the results are solely based on written voluntary feedback from self-selected patients who returned their feedback forms. To mitigate against this possible limitation, all patients who had received at least one visit from the prevention practitioner were also sent a copy of the patient feedback form and information letter in the post, along with a self-addressed, stamped envelope; this ensured that all participants had the opportunity to provide feedback, be it positive or not. Although this strategy could have resulted in patients submitting feedback more than once, the feedback received via mail-out was minimal.

A further limitation is that it is possible that patients who were interested in CDPS are overrepresented in the sample, as participation in the prevention visits was voluntary. Finally, as feedback was kept short and concise to respect patients’ time and mitigate against the demands associated with participating in BETTER 2, participants’ responses did not allow for extensive or more-elaborate analyses, and provided only a snapshot of patients’ perspectives.

### Comparison with existing literature

Although primary care providers often embody the role of patient advocate, patients’ own perspectives and identified needs often diverge from what providers assume to be patients’ best interests.^10^ Patients’ perceptions were in contrast with the perspectives of some doctors who participated in BETTER 2 and questioned the usefulness of the programme.^9^ Physicians perceived prevention visits to be a duplication of services already provided by their clinic, despite empirical evidence of a gap in preventive care, as demonstrated by the BETTER trial.^3^

Importantly, patients’ emphasis on the atmosphere of the visit, and the approachable and non-judgmental communication style used, is in accordance with emerging research that suggests that successful shifts in the patient and clinician relationship occur when a warm tone and climate are used;^11^ for instance, Bensing *et al* found that 'the emotional tone of the medical consultation seems to be very important from the patient perspective'.^10^

The collaborative approach used by the prevention practitioners in BETTER 2 involves the use of brief action planning,^6^ an approach based on the principles of shared decision making that enables patients to take ownership of their health while *collaboratively* determining next steps for prevention and screening. This also reflects a rising trend, in which primary care providers and patients work in an equal partnership.^11^ Although shared decision making has been promoted as a part of patient-centred care, uptake is still limited,^12,13^ despite evidence that it has positive effects on patients’ health.^14^

### Implications for research and practice

Although family physicians who see CDPS as an integral part of their practice may question the effectiveness of having a prevention practitioner in their primary care setting, patients’ feedback was overwhelmingly positive and they perceived their visits with a prevention practitioner as valuable and worthwhile. Patients expressed their desire to receive sustained CDPS in primary care, across urban and rural/remote contexts, which adds an important perspective on the impact and implementation of the BETTER approach. The findings presented here inform primary care teams who are interested in enhancing their CDPS practices.
